# Supplementary material for: Iron-Chelation Treatment by Novel Thiosemicarbazone Targets Major Signaling Pathways in Neuroblastoma
Source: Int J Mol Sci. 2021 Dec 29;23(1):376. doi: 10.3390/ijms23010376 (PMC8745636; doi:10.3390/ijms23010376)
Supplement: Supplementary file 1 [file ijms-23-00376-s001.zip › ijms-1485340-supplementary.pdf]

International Journal of *Molecular Sciences*

Article

Iron-chelation treatment by novel thiosemicarbazone targets major signaling pathways in neuroblastoma

Peter Macsek [1,2], Jan Skoda[1,2], Maria Krchniakova[1], Jakub Neradil [1,2,3,†,*] and Renata Veselska [1,2,3,†]

Supplementary materials

Table S1: Antibodies used in Western blot (WB), Immunofluorescence (IF) and Flow cytometry (FC) analyses.

Antigen	Source	Isotype	Dilution	Manufacturer	Cat. number
AKT	Rabbit	IgG	1:1000 (WB)	CST	4691
pAKT (S473)	Rabbit	IgG	1:2000 (WB)	CST	4060
C-MYC	Rabbit	IgG	1:1000 (WB)<br>1:100 (IF)	CST	5605
EGFR	Rabbit	IgG	1:1000 (WB)	CST	2646
pEGFR (Y845)	Rabbit	IgG	1:1000 (WB)	CST	6963
pEGFR (Y992)	Rabbit	IgG	1:1000 (WB)	CST	2235
pEGFR (Y1045)	Rabbit	IgG	1:1000 (WB)	CST	2237
pEGFR (Y1068)	Mouse	IgG	1:1000 (WB)	CST	2236
pEGFR (Y1148)	Rabbit	IgG	1:1000 (WB)	CST	4404
ERK 1/2	Rabbit	IgG	1:1000 (WB)	CST	4695
pERK 1/2 (T202/Y204)	Rabbit	IgG	1:1000 (WB)	CST	4370
GAPDH	Rabbit	IgG	1:10000 (WB)	CST	2118
HIF1A	Rabbit	IgG	1:400 (IF)	CST	36169
MIG-6	Rabbit	polyclonal	1:1000 (WB)	CST	2440
NDRG1	Rabbit	IgG	1:1000 (WB)<br>1:100 (IF)	CST	9485
pNDRG1 (T346)	Rabbit	IgG	1:2000 (WB)	CST	5482
N-MYC	Rabbit	IgG	1:1000 (WB)	CST	84406
HRP-conjugated anti-Rb	Goat	IgG	1:5000 (WB)	CST	7074
HRP-conjugated anti-Mo	Horse	IgG	1:5000 (WB)	CST	7076
Anti-Mouse IgG-Alexa Fluor® 488	Goat	IgG	1:200 (IF)	Invitrogen	A21202
Anti-Mouse IgG-Alexa Fluor® 568	Goat	IgG	1:200 (IF)	Invitrogen	A10037
Anti-Rabbit IgG-Alexa Fluor® 488	Goat	IgG	1:200 (IF)	Invitrogen	A21206
Anti-Rabbit IgG-Alexa Fluor® 568	Goat	IgG	1:200 (IF)	Invitrogen	A10042
pEGFR (Y1068)-Alexa Fluor® 488	Rabbit	IgG	1:100 (IF)<br>1:50 (FC)	Abcam	ab205827
IgG Isotype Ctrl-Alexa Fluor® 488	Rabbit	IgG	1:100 (IF)<br>1:50 (FC)	Abcam	ab199091

Table S2. Proteins detected by Proteome Profiler™ Human Cell Stress Array Kit.

**Proteome Profiler™ Human Cell Stress Array Kit**					
ADAMTS1	Bcl-2	CA9	Cited-2	COX-2	Cytochrome C
Dkk-4	FABP-1	HIF-1α	HIF-2α	p21/CIP1	HSP60
HSP70	IDO	Thioredoxin-1	NFκB1	PON3	p27/Kip1
SIRT2	SOD2	PON1	PON2	p53 (S46)	HSP27 (S78/S82)
p38α (T180/Y182)			JNK (T183/Y185)		

Table S3. Proteins detected by Proteome Profiler™ Human Phospho-Kinase Array Kit.

**Proteome Profiler™ Human Phospho-Kinase Array Kit**			
Protein	Phosphorylation	Protein	Phosphorylation
p38α	T180/Y182	STAT5a	Y689
ERK 1/2	T202/Y204, T185/Y187	p70 S6K^{II}	T421/S424
JNK 1/2/3	T183/Y185, T221/Y223	RSK 1/2/3	S380/S386/S377
GSK3-α/β	S21/S9	eNOS	S1177
p53^{I}	S392	Fyn	Y420
EGFR	Y1086	Yes	Y426
MSK 1/2	S376/S360	Fgr	Y412
AMPKα1	T183	STAT6	Y641
AKT 1/2/3^{I}	S473	STAT5b	Y699
AKT 1/2/3^{II}	T308	STAT3^{I}	Y705
p53^{II}	S46	p27	T198
TOR	S2448	PLC-γ1	Y783
CREB	S133	Hck	Y411
HSP27	S78/S82	Chk-2	T68
AMPKα2	T172	FAK	Y397
β-catenin	-	PDGFRβ	Y751
p70 S6K^{I}	T389	STAT5a/b	Y694/Y699
p53^{III}	S15	STAT3^{II}	S727
c-Jun	S63	WNK1	T60
Src	Y419	PYK2	Y402
Lyn	Y397	PRAS40	T246
Lck	Y394	HSP60	-
STAT2	Y689		

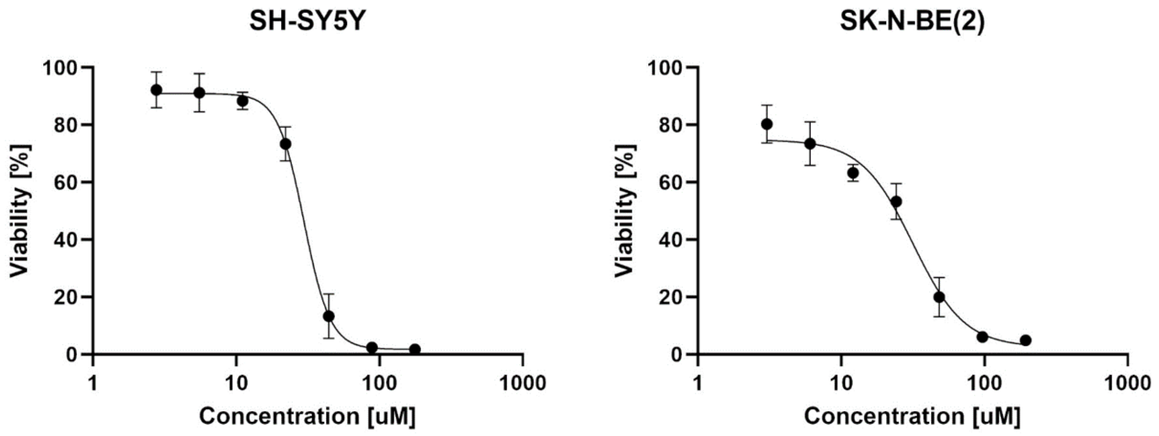

Figure S1: MTT cell viability assay in response to DpC treatment in SH-SY5Y and SK-N-BE(2) neuroblastoma cell lines

Methodology: The 3-[4,5-dimethylthiazol-2-yl]-2,5-diphenyltetrazolium bromide (MTT) assay was used to determine proliferation of cells after DpC treatment. The cells were seeded in 96-well plates at a density of 5x10^3 cells/well and were allowed to adhere overnight. The next day, medium was removed and replaced with fresh medium containing the appropriate concentrations of DpC. After 24-hour incubation under standard cell culture conditions, the cells were incubated with MTT (0.5 mg/ml; Sigma-Aldrich, St. Louis, MO, USA) for 3 hours at 37°C. Subsequently, the medium was removed, and formazan crystals were dissolved in 200 µl of DMSO. The absorbance was measured at 570 nm with a reference absorbance at 620 nm using a Sunrise Absorbance Reader (Tecan, Männedorf, Switzerland).

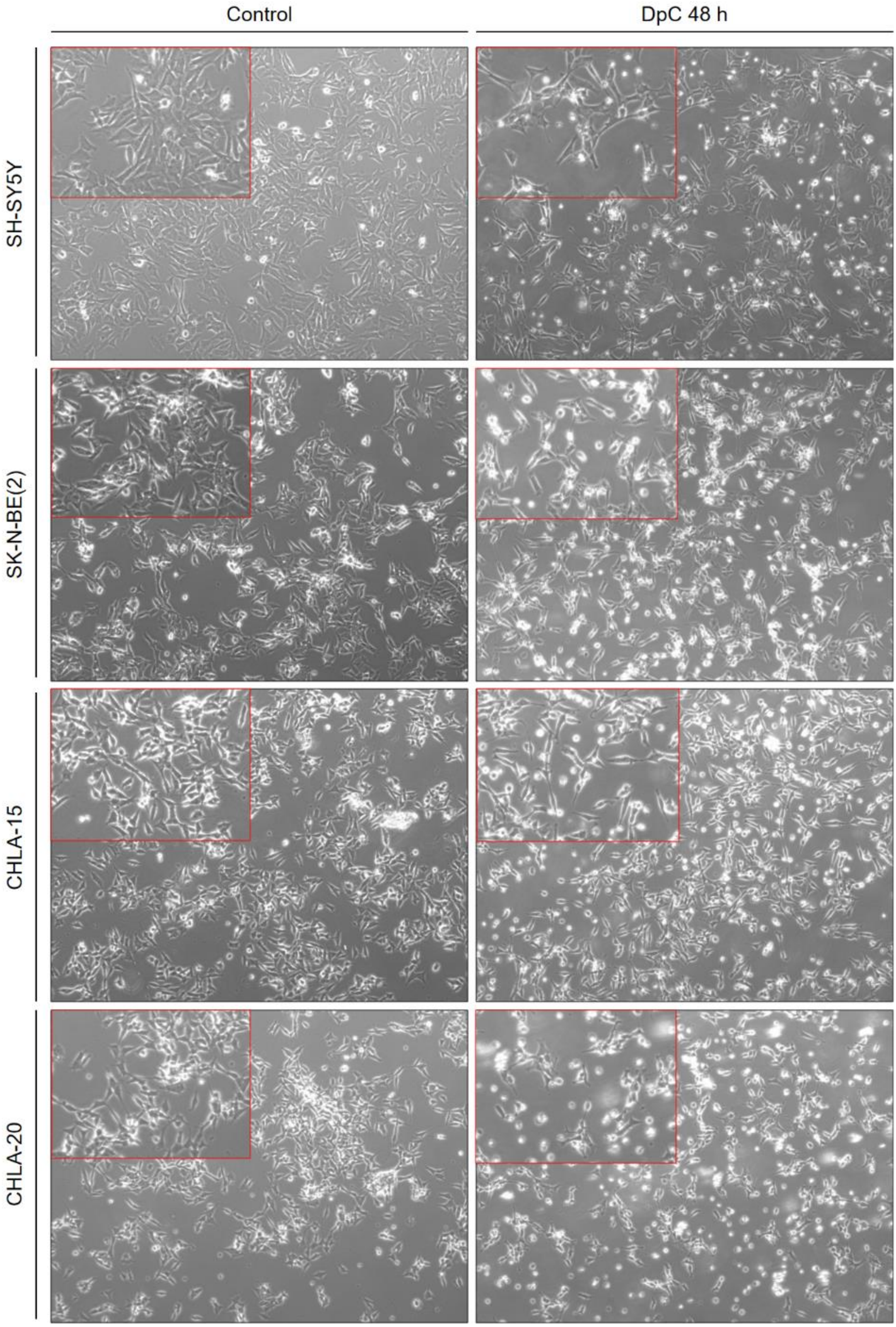

Figure S2: Bright field imaging of neuroblastoma cell lines in response to 20μM (SH-SY5Y, SK-N-BE(2)) or 2μM (CHLA-15, CHLA-20) DpC after 48 hours.

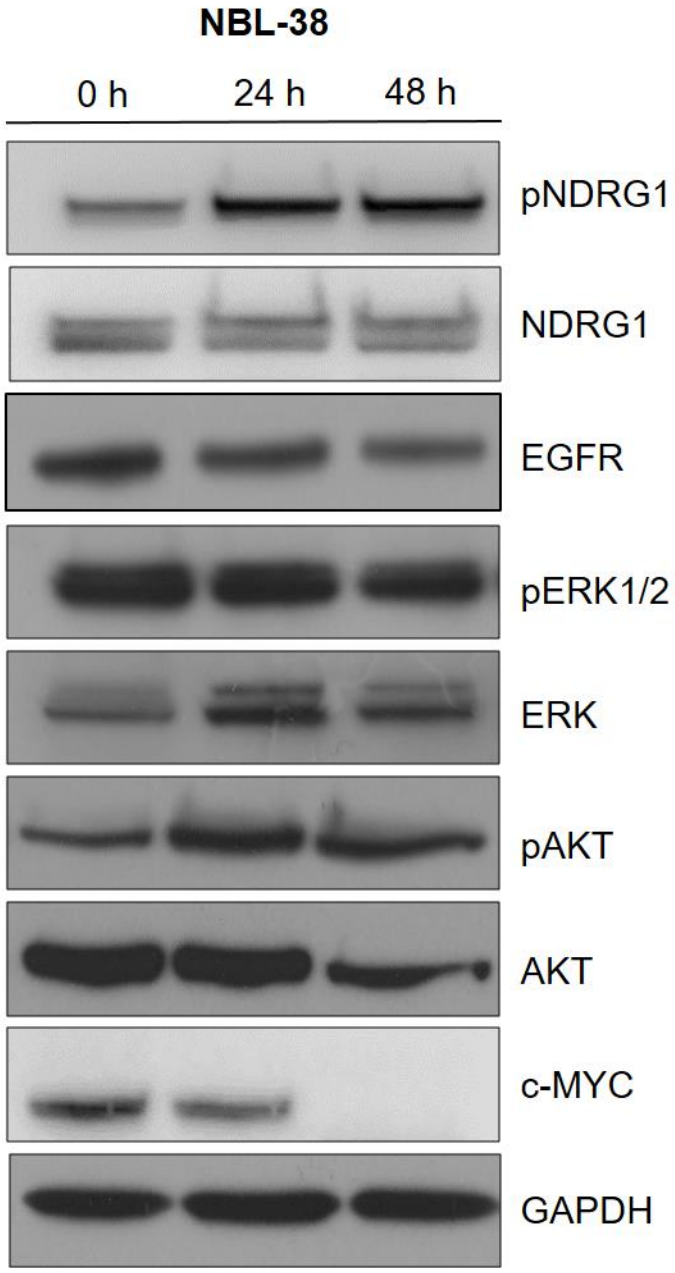

Figure. S3: Immunoblotting of various proteins regulated in response to 20μM DpC treatment for 24 and 48 hours in a primary human neuroblastoma cell line.

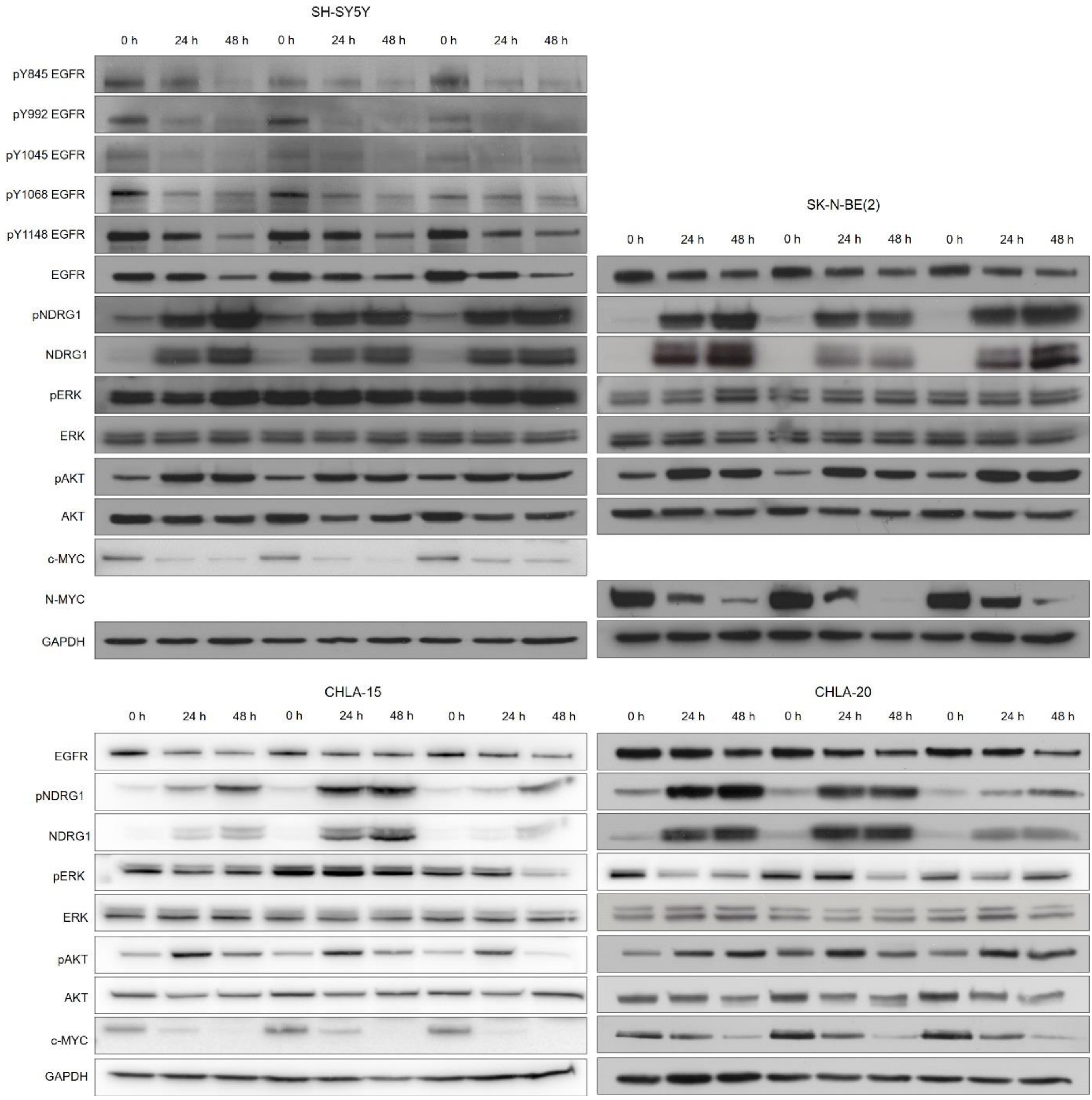

Figure S4: Western Blot analysis of individual biological triplicates of neuroblastoma cell lines treated with 20 µM (SH-SY5Y, SK-N-BE(2)) or 2 µM (CHLA-15, CHLA-20) DpC.

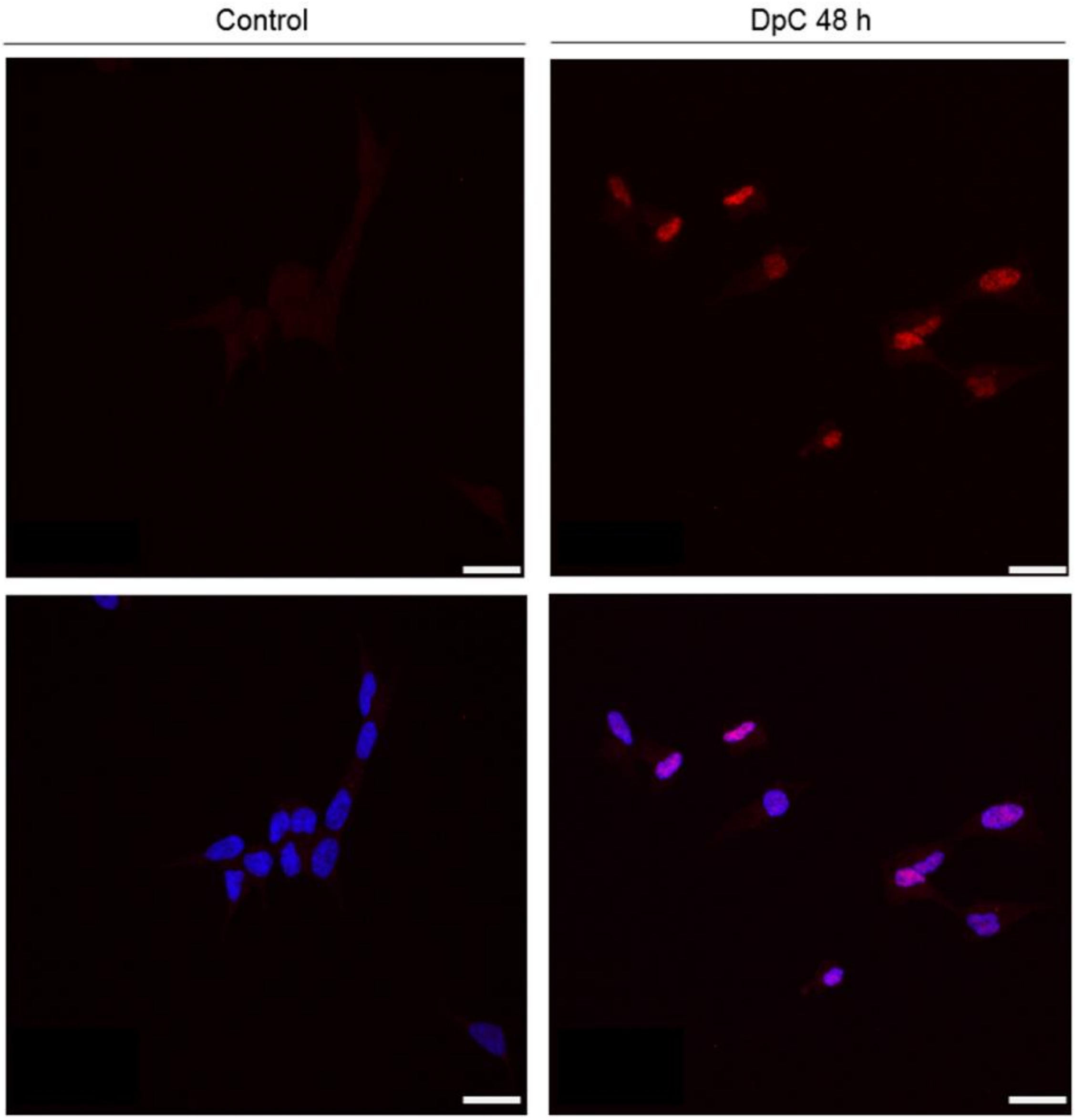

Figure S5: Immunofluorescence micrograph of HIF1A (red) in SH-SY5Y cells treated with 20 μM DpC. Nuclei counter-stained with Hoechst 33342 (blue). Scalebar = 20 μm.
